# 2-(7-Hydr­oxy-2-naphth­yloxy)-*N*-(6-methyl-2-pyrid­yl)acetamide

**DOI:** 10.1107/S1600536808006211

**Published:** 2008-03-12

**Authors:** Hoong-Kun Fun, Samuel Robinson Jebas, Subrata Jana, Rinku Chakrabarty, Shyamaprasad Goswami

**Affiliations:** a X-ray Crystallography Unit, School of Physics, Universiti Sains Malaysia, 11800 USM, Penang, Malaysia; b Department of Chemistry, Bengal Engineering and Science University, Shibpur, Howrah 711 103, India

## Abstract

In the title compound, C_18_H_16_N_2_O_3_, the dihedral angle between the naphthalene ring system and the pyridyl ring is 18.1 (8)°. The mol­ecules are inter­connected *via* C—H⋯O and O—H⋯O hydrogen bonds. Inversion-related mol­ecules are linked by O—H⋯O hydrogen bonds into cyclic centrosymmetric *R*_2_^2^(22) dimers. Intra­molecular N—H⋯O hydrogen bonding produces an *S*(5) ring motif. The crystal structure is further stabilized by weak C—H—π inter­actions.

## Related literature

For related literature on the applications; see: Atwood *et al.* (1996 ▶); Garcia-Tellado *et al.* (1990 ▶); Ghosh & Masanta (2006 ▶). For comparison bond lengths and angles see: Jin & Jin (2005 ▶); Liu & Li (2004 ▶); Rozycka-Sokolowska *et al.* (2004 ▶).
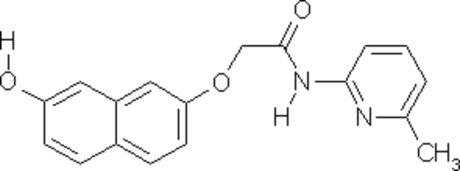


## Experimental

### 

#### Crystal data

C_18_H_16_N_2_O_3_
*M* *_r_* = 308.33
Triclinic, 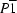

*a* = 5.3676 (3) Å
*b* = 11.6991 (7) Å
*c* = 12.2915 (6) Å
α = 104.994 (4)°
β = 94.777 (3)°
γ = 94.877 (4)°
*V* = 738.42 (7) Å^3^
*Z* = 2
Mo *K*α radiation
μ = 0.10 mm^−1^
*T* = 100.0 (1) K
0.4 × 0.16 × 0.09 mm

#### Data collection

Bruker SMART APEXII CCD area-detector diffractometer
Absorption correction: multi-scan (*SADABS*; Bruker, 2005 ▶) *T*_min_ = 0.963, *T*_max_ = 0.992
12299 measured reflections
3340 independent reflections
2480 reflections with *I* > 2σ(*I*)
*R*_int_ = 0.046

#### Refinement

*R*[*F* ^2^ > 2σ(*F* ^2^)] = 0.045
*wR*(*F* ^2^) = 0.130
*S* = 1.08
3340 reflections
217 parameters
H atoms treated by a mixture of independent and constrained refinement
Δρ_max_ = 0.24 e Å^−3^
Δρ_min_ = −0.31 e Å^−3^

### 

Data collection: *APEX2* (Bruker, 2005 ▶); cell refinement: *APEX2*; data reduction: *SAINT* (Bruker, 2005 ▶); program(s) used to solve structure: *SHELXTL* (Sheldrick, 2008 ▶); program(s) used to refine structure: *SHELXTL*; molecular graphics: *SHELXTL*; software used to prepare material for publication: *SHELXTL* and *PLATON* (Spek, 2003 ▶).

## Supplementary Material

Crystal structure: contains datablocks global, I. DOI: 10.1107/S1600536808006211/ng2430sup1.cif

Structure factors: contains datablocks I. DOI: 10.1107/S1600536808006211/ng2430Isup2.hkl

Additional supplementary materials:  crystallographic information; 3D view; checkCIF report

## Figures and Tables

**Table 1:** Hydrogen-bond geometry (Å, °)

*D*—H⋯*A*	*D*—H	H⋯*A*	*D*⋯*A*	*D*—H⋯*A*
C11—H11*B*⋯O3^i^	0.97	2.45	3.410 (2)	168
N1—H1*N*1⋯O1	0.88 (2)	2.11 (2)	2.5688 (18)	111.9 (16)
O3—H1*O*3⋯O2^ii^	0.88 (3)	1.85 (2)	2.6575 (17)	152 (2)
C11—H11*A*⋯*Cg*1^iii^	0.97	2.63	3.438	141
C18—H18*A*⋯*Cg*2^iv^	0.97	2.92	3.805	153

Symmetry codes: (i) 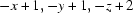; (ii) 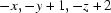; (iii) 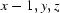; (iv) 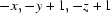. *Cg*1 is the centroid of the C1,C2,C7–C10 ring and *Cg*2 is the centroid of C2–C7 ring.

**Fig. 1.**
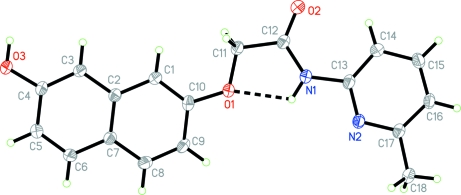
The molecular structure of the title compound, showing 50% probability displacement ellipsoids and the atomic numbering scheme. Hydrogen bonds are shown as dashed lines.

**Fig. 2.**
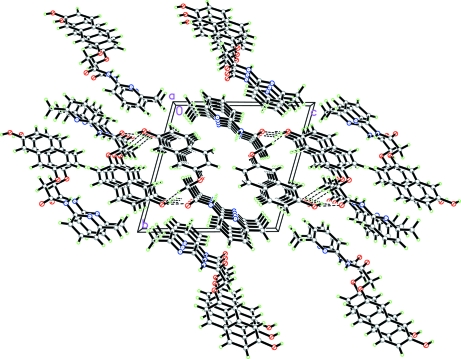
The crystal packing of the title compound, viewed along the a axis. Hydrogen bonds are shown as dashed lines.

## supplementary crystallographic information

### Comment

Pyridine amide moiety is widely used for the recognition of carboxylic acid functional group due to its complementary donor-acceptor arrangement (Garcia-Tellado *et al.*, 1990). This group attached with different spacer having photo physical properties is the current interest for the recognition studies of both mono/di carboxylic acids (Ghosh & Masanta, 2006).This type of compounds is also important for its unique supramolecular arrangement (Atwood *et al.*, 1996).

The asymmetric unit of (I) contains one molecule of 2–(7-hydroxy– naphthalene-2-yloxy)-*N*-(6-methyl-pyridine–2–yl) –acetamide. The dihedral angle between the naphthalene ring and the pyridine rings being 18.03 (8)°. The bond lengths and bond angles are comparable with the values reported in the literature (Rozycka-Sokolowska *et al.*, 2004; Jin & Jin, 2005). The bond distance of C12=O2 is 1.226 (2) Å, which is typical for double bonds (Liu & Li., 2004). The naphthalene ring is planar, the maximum deviation from the least squares plane being -0.011 (2) Å for atom C10. The pyridine ring is planar with the maximum deviation from planarity being -0.010 (2) Å for atom C17.

The molecules are stacked into layers parallel to the *bc*-plane by C11—H11B—O3^i^ and O3—H1O3—O2^ii^ hydrogen bonds (Fig. 2). In the crystal structure of (I), inversion-related molecules at (*x*,*y*,*z*) and (2 - *x*,1 - *y*,3 - *z*) are linked by O3—H1O3—O2 hydrogen bonds into cyclic centrosymmetric *R*_2_^2^(22) dimers. The crystal structure is further stabilized by weak C—H—π interactions involving rings C11—H11A—*Cg*1 (where *Cg*1 is the centroid of the C1,C2,C7—C10 ring) and C18—H18A—*Cg*2 (where *Cg*2 is the centroid of C2—C7 ring). The molecular conformation is stabilized by a N1—H1N1—O1 intramolecular interaction generating a ring motif S(5).

### Experimental

2,7-Dihydroxynaphthalene (160 mg, 1.0 mmol) and *N*–picolylchloroacetamide (185 mg, 1.0 mmol) were stirred with K_2_CO_3_ (345 mg, 2.5 mmol) and *^t^*Bu4N^+^Br^-^ (50 mg, 0.16 mmol) in dry acetone (10 ml) for 7 h at room temperature. Acetone was then distilled off and the crude product was extracted with CHCl_3_ (4 *x* 20 ml) after washing with water. The product (I) was purified by column chromatography (Silica gel 100–200 mesh) using 20% ethyl acetate in pet ether as eluent to afford an off-white coloured solid compound (Yield 61%). Single crystals were grown by slow evaporation of CHCl_3_/MeOH/Xylene solution (*v*/*v* 1:1:3) (Mp. 178–80 °C).

### Refinement

H atoms were placed in calculated positions, with C—H=0.93 Å,and O—H=0.86 Å, N—H=0.86 Å, and refined using a riding model, with *U*_iso_(H)=1.2U_equ_(C,*N*,*O*).

### Crystal data

**Table:**

C_18_H_16_N_2_O_3_	*Z* = 2
*M**_r_* = 308.33	*F*_000_ = 324
Triclinic, *P*1	*D*_x_ = 1.387 Mg m^−^^3^
Hall symbol: -P 1	Mo *K*α radiation λ = 0.71073 Å
*a* = 5.3676 (3) Å	Cell parameters from 2511 reflections
*b* = 11.6991 (7) Å	θ = 3.4–30.4º
*c* = 12.2915 (6) Å	µ = 0.10 mm^−^^1^
α = 104.994 (4)º	*T* = 100.0 (1) K
β = 94.777 (3)º	Block, colourless
γ = 94.877 (4)º	0.4 × 0.16 × 0.09 mm
*V* = 738.42 (7) Å^3^	

### Data collection

**Table:**

Bruker SMART APEXII CCD area-detector diffractometer	2480 reflections with *I* > 2σ(*I*)
Detector resolution: 8.33 pixels mm^-1^	*R*_int_ = 0.046
*T* = 100.0(1) K	θ_max_ = 27.5º
ω scans	θ_min_ = 1.7º
Absorption correction: multi-scan(SADABS; Bruker, 2005)	*h* = −6→6
*T*_min_ = 0.963, *T*_max_ = 0.992	*k* = −15→13
12299 measured reflections	*l* = −15→15
3340 independent reflections	

### Refinement

**Table:**

Refinement on *F*^2^	H atoms treated by a mixture of independent and constrained refinement
Least-squares matrix: full	*w* = 1/[σ^2^(*F*_o_^2^) + (0.0577*P*)^2^ + 0.2187*P*] where *P* = (*F*_o_^2^ + 2*F*_c_^2^)/3
*R*[*F*^2^ > 2σ(*F*^2^)] = 0.045	(Δ/σ)_max_ < 0.001
*wR*(*F*^2^) = 0.130	Δρ_max_ = 0.24 e Å^−^^3^
*S* = 1.08	Δρ_min_ = −0.31 e Å^−^^3^
3340 reflections	Extinction correction: none
217 parameters	

### Special details

**Table:**

Geometry. Experimental. The low-temperature data was collected with the Oxford Crysosystem Cobra low-temperature attachement.All e.s.d.'s (except the e.s.d. in the dihedral angle between two l.s. planes) are estimated using the full covariance matrix. The cell e.s.d.'s are taken into account individually in the estimation of e.s.d.'s in distances, angles and torsion angles; correlations between e.s.d.'s in cell parameters are only used when they are defined by crystal symmetry. An approximate (isotropic) treatment of cell e.s.d.'s is used for estimating e.s.d.'s involving l.s. planes.

### Fractional atomic coordinates and isotropic or equivalent isotropic displacement parameters (Å^2^)

**Table:**

	*x*	*y*	*z*	*U*_iso_*/*U*_eq_	
O1	−0.0438 (2)	0.40705 (11)	0.65093 (10)	0.0240 (3)	
O2	−0.5889 (2)	0.23059 (11)	0.67844 (10)	0.0252 (3)	
O3	0.8081 (2)	0.77389 (11)	1.13643 (11)	0.0245 (3)	
N1	−0.3537 (3)	0.23114 (14)	0.53179 (13)	0.0225 (3)	
N2	−0.3711 (3)	0.13147 (13)	0.34601 (12)	0.0204 (3)	
C1	0.2212 (3)	0.52560 (15)	0.81993 (14)	0.0202 (4)	
H1A	0.1222	0.4975	0.8681	0.024*	
C2	0.4376 (3)	0.60921 (15)	0.86540 (14)	0.0188 (4)	
C3	0.5095 (3)	0.65141 (15)	0.98390 (14)	0.0201 (4)	
H3A	0.4128	0.6258	1.0342	0.024*	
C4	0.7222 (3)	0.73013 (15)	1.02417 (14)	0.0199 (4)	
C5	0.8699 (3)	0.77091 (16)	0.94921 (15)	0.0222 (4)	
H5A	1.0129	0.8245	0.9777	0.027*	
C6	0.8034 (3)	0.73183 (15)	0.83490 (15)	0.0220 (4)	
H6A	0.9019	0.759	0.786	0.026*	
C7	0.5856 (3)	0.65024 (15)	0.79020 (14)	0.0194 (4)	
C8	0.5140 (3)	0.60795 (16)	0.67167 (15)	0.0218 (4)	
H8A	0.6104	0.6349	0.622	0.026*	
C9	0.3063 (3)	0.52862 (16)	0.62984 (15)	0.0219 (4)	
H9A	0.2608	0.5019	0.5521	0.026*	
C10	0.1598 (3)	0.48687 (15)	0.70512 (15)	0.0212 (4)	
C11	−0.2068 (3)	0.35993 (16)	0.71767 (15)	0.0214 (4)	
H11A	−0.2874	0.4234	0.7641	0.026*	
H11B	−0.111	0.3235	0.7675	0.026*	
C12	−0.4042 (3)	0.26785 (15)	0.64022 (14)	0.0201 (4)	
C13	−0.4891 (3)	0.14402 (15)	0.43903 (15)	0.0206 (4)	
C14	−0.7171 (3)	0.08120 (16)	0.44454 (16)	0.0241 (4)	
H14A	−0.7945	0.0941	0.511	0.029*	
C15	−0.8235 (3)	−0.00193 (17)	0.34564 (16)	0.0259 (4)	
H15A	−0.9753	−0.0469	0.3448	0.031*	
C16	−0.7042 (3)	−0.01777 (16)	0.24877 (15)	0.0227 (4)	
H16A	−0.7733	−0.0742	0.1826	0.027*	
C17	−0.4792 (3)	0.05155 (15)	0.25079 (14)	0.0198 (4)	
C18	−0.3482 (3)	0.04513 (16)	0.14692 (15)	0.0244 (4)	
H18D	−0.1719	0.0416	0.1646	0.037*	
H18A	−0.3712	0.1145	0.1209	0.037*	
H18B	−0.4174	−0.0248	0.0887	0.037*	
H1N1	−0.210 (4)	0.2626 (18)	0.5165 (17)	0.026 (5)*	
H1O3	0.700 (5)	0.756 (2)	1.181 (2)	0.043 (7)*	

### Atomic displacement parameters (Å^2^)

**Table:**

	*U*^11^	*U*^22^	*U*^33^	*U*^12^	*U*^13^	*U*^23^
O1	0.0247 (6)	0.0271 (7)	0.0162 (6)	−0.0067 (5)	0.0001 (5)	0.0022 (5)
O2	0.0267 (6)	0.0274 (7)	0.0193 (7)	−0.0016 (5)	0.0039 (5)	0.0034 (5)
O3	0.0262 (6)	0.0291 (7)	0.0146 (7)	−0.0054 (5)	−0.0010 (5)	0.0031 (5)
N1	0.0234 (7)	0.0243 (8)	0.0165 (8)	−0.0042 (6)	0.0005 (6)	0.0021 (6)
N2	0.0226 (7)	0.0195 (7)	0.0174 (8)	−0.0006 (5)	−0.0010 (6)	0.0037 (6)
C1	0.0224 (8)	0.0214 (9)	0.0162 (9)	0.0007 (6)	0.0028 (7)	0.0042 (7)
C2	0.0210 (8)	0.0170 (8)	0.0174 (9)	0.0027 (6)	0.0020 (7)	0.0025 (7)
C3	0.0233 (8)	0.0205 (9)	0.0161 (9)	0.0001 (6)	0.0028 (7)	0.0048 (7)
C4	0.0233 (8)	0.0186 (9)	0.0159 (9)	0.0019 (6)	−0.0002 (7)	0.0020 (7)
C5	0.0219 (8)	0.0213 (9)	0.0211 (10)	−0.0015 (6)	0.0010 (7)	0.0035 (7)
C6	0.0238 (8)	0.0210 (9)	0.0207 (9)	−0.0011 (7)	0.0047 (7)	0.0051 (7)
C7	0.0231 (8)	0.0177 (8)	0.0168 (9)	0.0021 (6)	0.0022 (7)	0.0035 (7)
C8	0.0258 (8)	0.0216 (9)	0.0187 (9)	0.0034 (7)	0.0050 (7)	0.0058 (7)
C9	0.0281 (9)	0.0226 (9)	0.0133 (9)	0.0028 (7)	0.0008 (7)	0.0021 (7)
C10	0.0219 (8)	0.0182 (9)	0.0203 (9)	0.0021 (6)	−0.0012 (7)	0.0004 (7)
C11	0.0239 (8)	0.0219 (9)	0.0167 (9)	0.0009 (7)	0.0003 (7)	0.0031 (7)
C12	0.0241 (8)	0.0196 (9)	0.0158 (9)	0.0027 (7)	−0.0001 (7)	0.0038 (7)
C13	0.0241 (8)	0.0195 (9)	0.0165 (9)	0.0008 (6)	−0.0013 (7)	0.0033 (7)
C14	0.0251 (9)	0.0277 (10)	0.0186 (9)	−0.0014 (7)	0.0022 (7)	0.0062 (8)
C15	0.0240 (8)	0.0277 (10)	0.0241 (10)	−0.0060 (7)	−0.0029 (7)	0.0082 (8)
C16	0.0257 (8)	0.0205 (9)	0.0183 (9)	−0.0032 (7)	−0.0038 (7)	0.0028 (7)
C17	0.0233 (8)	0.0183 (8)	0.0168 (9)	0.0022 (6)	−0.0012 (7)	0.0039 (7)
C18	0.0272 (9)	0.0243 (9)	0.0183 (9)	0.0000 (7)	−0.0001 (7)	0.0016 (7)

### Geometric parameters (Å, °)

**Table:**

O1—C10	1.376 (2)	C6—H6A	0.93
O1—C11	1.419 (2)	C7—C8	1.420 (2)
O2—C12	1.226 (2)	C8—C9	1.360 (2)
O3—C4	1.366 (2)	C8—H8A	0.93
O3—H1O3	0.88 (3)	C9—C10	1.415 (3)
N1—C12	1.348 (2)	C9—H9A	0.93
N1—C13	1.415 (2)	C11—C12	1.516 (2)
N1—H1N1	0.88 (2)	C11—H11A	0.97
N2—C13	1.334 (2)	C11—H11B	0.97
N2—C17	1.345 (2)	C13—C14	1.389 (2)
C1—C10	1.368 (2)	C14—C15	1.388 (2)
C1—C2	1.426 (2)	C14—H14A	0.93
C1—H1A	0.93	C15—C16	1.377 (3)
C2—C7	1.415 (2)	C15—H15A	0.93
C2—C3	1.420 (2)	C16—C17	1.391 (2)
C3—C4	1.374 (2)	C16—H16A	0.93
C3—H3A	0.93	C17—C18	1.496 (3)
C4—C5	1.411 (2)	C18—H18D	0.96
C5—C6	1.366 (2)	C18—H18A	0.96
C5—H5A	0.93	C18—H18B	0.96
C6—C7	1.418 (2)		
			
C10—O1—C11	118.54 (13)	C1—C10—O1	125.35 (16)
C4—O3—H1O3	112.9 (16)	C1—C10—C9	121.31 (16)
C12—N1—C13	129.91 (15)	O1—C10—C9	113.33 (15)
C12—N1—H1N1	115.8 (13)	O1—C11—C12	109.16 (14)
C13—N1—H1N1	114.2 (13)	O1—C11—H11A	109.8
C13—N2—C17	117.79 (14)	C12—C11—H11A	109.8
C10—C1—C2	119.74 (17)	O1—C11—H11B	109.8
C10—C1—H1A	120.1	C12—C11—H11B	109.8
C2—C1—H1A	120.1	H11A—C11—H11B	108.3
C7—C2—C3	119.23 (15)	O2—C12—N1	125.19 (16)
C7—C2—C1	119.02 (15)	O2—C12—C11	120.00 (15)
C3—C2—C1	121.74 (16)	N1—C12—C11	114.78 (15)
C4—C3—C2	119.93 (16)	N2—C13—C14	124.72 (16)
C4—C3—H3A	120	N2—C13—N1	111.27 (15)
C2—C3—H3A	120	C14—C13—N1	124.00 (17)
O3—C4—C3	124.03 (16)	C15—C14—C13	116.46 (17)
O3—C4—C5	115.14 (15)	C15—C14—H14A	121.8
C3—C4—C5	120.83 (16)	C13—C14—H14A	121.8
C6—C5—C4	120.17 (15)	C16—C15—C14	120.03 (16)
C6—C5—H5A	119.9	C16—C15—H15A	120
C4—C5—H5A	119.9	C14—C15—H15A	120
C5—C6—C7	120.59 (17)	C15—C16—C17	119.32 (16)
C5—C6—H6A	119.7	C15—C16—H16A	120.3
C7—C6—H6A	119.7	C17—C16—H16A	120.3
C2—C7—C6	119.24 (15)	N2—C17—C16	121.65 (16)
C2—C7—C8	119.26 (15)	N2—C17—C18	116.05 (15)
C6—C7—C8	121.50 (16)	C16—C17—C18	122.26 (15)
C9—C8—C7	120.91 (17)	C17—C18—H18D	109.5
C9—C8—H8A	119.5	C17—C18—H18A	109.5
C7—C8—H8A	119.5	H18D—C18—H18A	109.5
C8—C9—C10	119.75 (16)	C17—C18—H18B	109.5
C8—C9—H9A	120.1	H18D—C18—H18B	109.5
C10—C9—H9A	120.1	H18A—C18—H18B	109.5
			
C10—C1—C2—C7	−0.2 (2)	C11—O1—C10—C9	−179.27 (14)
C10—C1—C2—C3	−179.63 (16)	C8—C9—C10—C1	0.7 (3)
C7—C2—C3—C4	−0.7 (3)	C8—C9—C10—O1	−179.89 (15)
C1—C2—C3—C4	178.70 (16)	C10—O1—C11—C12	−175.79 (14)
C2—C3—C4—O3	−178.86 (15)	C13—N1—C12—O2	−1.0 (3)
C2—C3—C4—C5	0.6 (3)	C13—N1—C12—C11	177.16 (16)
O3—C4—C5—C6	179.21 (16)	O1—C11—C12—O2	−167.56 (15)
C3—C4—C5—C6	−0.3 (3)	O1—C11—C12—N1	14.2 (2)
C4—C5—C6—C7	0.1 (3)	C17—N2—C13—C14	−0.6 (3)
C3—C2—C7—C6	0.5 (2)	C17—N2—C13—N1	−179.81 (14)
C1—C2—C7—C6	−178.93 (15)	C12—N1—C13—N2	−178.05 (16)
C3—C2—C7—C8	179.92 (16)	C12—N1—C13—C14	2.7 (3)
C1—C2—C7—C8	0.5 (2)	N2—C13—C14—C15	1.4 (3)
C5—C6—C7—C2	−0.2 (3)	N1—C13—C14—C15	−179.45 (16)
C5—C6—C7—C8	−179.62 (16)	C13—C14—C15—C16	−0.6 (3)
C2—C7—C8—C9	−0.2 (3)	C14—C15—C16—C17	−1.0 (3)
C6—C7—C8—C9	179.21 (16)	C13—N2—C17—C16	−1.1 (2)
C7—C8—C9—C10	−0.4 (3)	C13—N2—C17—C18	176.69 (15)
C2—C1—C10—O1	−179.74 (15)	C15—C16—C17—N2	1.9 (3)
C2—C1—C10—C9	−0.4 (3)	C15—C16—C17—C18	−175.75 (17)
C11—O1—C10—C1	0.2 (2)		

### Hydrogen-bond geometry (Å, °)

**Table:**

*D*—H···*A*	*D*—H	H···*A*	*D*···*A*	*D*—H···*A*
C11—H11B···O3^i^	0.97	2.45	3.410 (2)	168
N1—H1N1···O1	0.88 (2)	2.11 (2)	2.5688 (18)	111.9 (16)
O3—H1O3···O2^ii^	0.88 (3)	1.85 (2)	2.6575 (17)	152 (2)
C11—H11A···Cg1^iii^	0.97	2.63	3.438	141
C18—H18A···Cg2^iv^	0.97	2.93	3.805	153

Symmetry codes: (i) −*x*+1, −*y*+1, −*z*+2; (ii) −*x*, −*y*+1, −*z*+2; (iii) *x*−1, *y*, *z*; (iv) −*x*, −*y*+1, −*z*+1.
